# Complete Genomic RNA Sequence of Tuberose Mild Mosaic Virus and Tuberose Mild Mottle Virus Acquired by High-Throughput Sequencing

**DOI:** 10.3390/pathogens11080861

**Published:** 2022-07-30

**Authors:** Malyaj R. Prajapati, Aakansha Manav, Pankhuri Singhal, Venkidusamy K. Sidharthan, Ujjwal Sirohi, Mukesh Kumar, Mahesh Kumar Bharti, Jitender Singh, Pankaj Kumar, Ravindra Kumar, Satya Prakash, Virendra Kumar Baranwal

**Affiliations:** 1 College of Biotechnology, Sardar Vallabhbhai Patel University of Agriculture and Technology, Meerut 250110, Uttar Pradesh, India; malyajrprajapati@gmail.com (M.R.P.); aakanshasingh721@gmail.com (A.M.); panks.svpuat@gmail.com (P.K.); kumarrk2000@yahoo.com (R.K.); 2 Division of Plant Pathology, ICAR-Indian Agricultural Research Institute, New Delhi 110012, India; pankhuri.agri47@gmail.com (P.S.); vbaranwal2001@yahoo.com (V.K.B.); 3 Division of Genetics and Tree Improvement, Institute of Forest Biodiversity (ICFRE), Hyderabad 500100, Telangana, India; kavisidharthan.v@gmail.com; 4 College of Agriculture, Sardar Vallabhbhai Patel University of Agriculture and Technology, Meerut 250110, Uttar Pradesh, India; sirohiujjwal@gmail.com; 5 College of Horticulture, Sardar Vallabhbhai Patel University of Agriculture and Technology, Meerut 250110, Uttar Pradesh, India; k.mukesh123@yahoo.com (M.K.); satyaagro@gmail.com (S.P.); 6 College of Veterinary and Animal Science, Sardar Vallabhbhai Patel University of Agriculture and Technology, Meerut 250110, Uttar Pradesh, India; bhartibiochem@gmail.com

**Keywords:** tuberose, TuMMV, TuMMoV, high-throughput sequencing, phylogenetic analysis

## Abstract

Tuberose (*Polianthes tuberosa*) is an ornamental flowering crop of the *Amaryllidaceae* family. Tuberose mild mosaic virus (TuMMV) and tuberose mild mottle virus (TuMMoV), members of the genus *Potyvirus*, are ubiquitously distributed in most tuberose growing countries worldwide with low biological incidence. Here, we report the first coding-complete genomic RNA of TuMMV and TuMMoV obtained through high-throughput sequencing (HTS) and further, the presence of both the viruses were confirmed using virus-specific primers in RT-PCR assays. Excluding the poly (A) tail, the coding-complete genomic RNA of TuMMV and TuMMoV was 9485 and 9462 nucleotides (nts) in length, respectively, and contained a single large open reading frame (ORF). Polyprotein encoded by both the viral genomes contained nine putative cleavage sites. BLASTn analysis of TuMMV and TuMMoV genomes showed 72.40–76.80% and 67.95–77% nucleotide sequence similarities, respectively, with the existing potyviral sequences. Phylogenetic analysis based on genome sequences showed that TuMMV and TuMMoV clustered in a distinct clade to other potyviruses. Further studies are required to understand the mechanism of symptom development, distribution, genetic variability, and their possible threat to tuberose production in India.

## 1. Introduction

*Polianthes tuberosa*, also known as tuberose, is an important perennial herb within *Amaryllidaceae* family. In the country’s tropical regions, it is frequently grown as an ornamental blooming plant for the development of long-lasting flower spikes. It is also known as “Rajanigandha” or “Nishigandha” in popular parlance. It is believed to have originated in Mexico. In India, it is popularly grown in Andhra Pradesh, Assam, Gujarat, Karnataka, Punjab, Rajasthan, Tamil Nadu, West Bengal, and parts of Uttar Pradesh. It is rich in fragrance constituents as well as secondary metabolites, including polyphenols, nonpolar volatiles, and benzenoid derivatives. Furthermore, the flower and bulb extracts exhibit anti-inflammatory, antispasmodic, diuretic, and emetic effects [1].

Potyviridae contains about 200 plant virus species that are now allocated to the twelve genera Arepavirus, Bevemovirus, Brambyvirus, Bymovirus, Celavirus, Ipomovirus, Macluravirus, Poacevirus, Roymovirus, Rymovirus, Tritimovirus, and Potyvirus [2]. Members of the family are distinguished by host range, vector, genomic features, and phylogeny, with species demarcation frequently based on sequence identity of the main ORF (or, if necessary, the CP-coding region) being <76% (nt) and <82% (aa) [3].

*Potyviridae* members can be monopartite or bipartite (flexuous filamentous particles 650–950 nm long and 11–20 nm wide), single-stranded positive-sense RNA genomes with sizes ranging from 8.2 kb to 11.3 kb, with an average size of 9.7 kb. Genomic RNA is translated into polyproteins that must be proteolytically processed to provide ten mature and one fusion protein required for replication and motility: P1 (modulator of replication and translation), helper component proteinase HC-Pro (aphid transmission and silencing suppression), P3 (movement virus and replication), P3N-PIPO (cell-to-cell movement), 6K1 (formation of replication vesicles), cytoplasmic inclusion, CI (replication and helicase involved in virus movement), 6K2 (formation of replication vesicles), genome-linked protein VPg (replication, translation, and virus movement), NIa-Pro (polyprotein processing), NIb (RNA-dependent RNA polymerase), and CP (aphid transmission, virion formation and virus movement) [3].

Some members have a narrow host range, whilst others can infect a wide range of hosts [4]. Several of the members cause disease symptoms in commercially significant crops, rendering this genus one of the largest thoroughly researched virus groups by plant virologists. Potyviruses are primarily spread by aphids in a non-persistent manner as well as by mechanical means. Vertical transmission of potyviruses through seeds has also been documented [5]. Considering the economic importance of tuberose, identifying and characterizing the disease-causing viruses of tuberose is a prerequisite for devising suitable management strategies.

The rapid development of high-throughput sequencing (HTS) technology in recent years has not only enabled the investigation of diseases with unknown etiology but also facilitated the identification and characterization of known/novel viruses [6,7,8,9,10]. Unlike traditional antibody and nucleic acid-based detection techniques, such as enzyme-linked immunosorbent assay (ELISA), hybridization and PCR assays, HTS technologies do not warrant any virus-specific primers/probes/antibodies for detection of target pathogens. Through bioinformatic analysis of HTS reads, viruses associated with a particular sample can be unveiled and their genomes be recovered [11]. Besides, HTS facilitates detection of multiple infection of a plant species, thereby, helping us to comprehensively capture the entire virome and viral variability [8,9,10,11,12]. Using HTS technology, this study reports the first coding-complete genomic RNA sequence of tuberose mild mosaic virus (TuMMV) and tuberose mild mottle virus (TuMMoV) from diseased samples in India.

## 2. Results and Discussion

**Figure 1:**
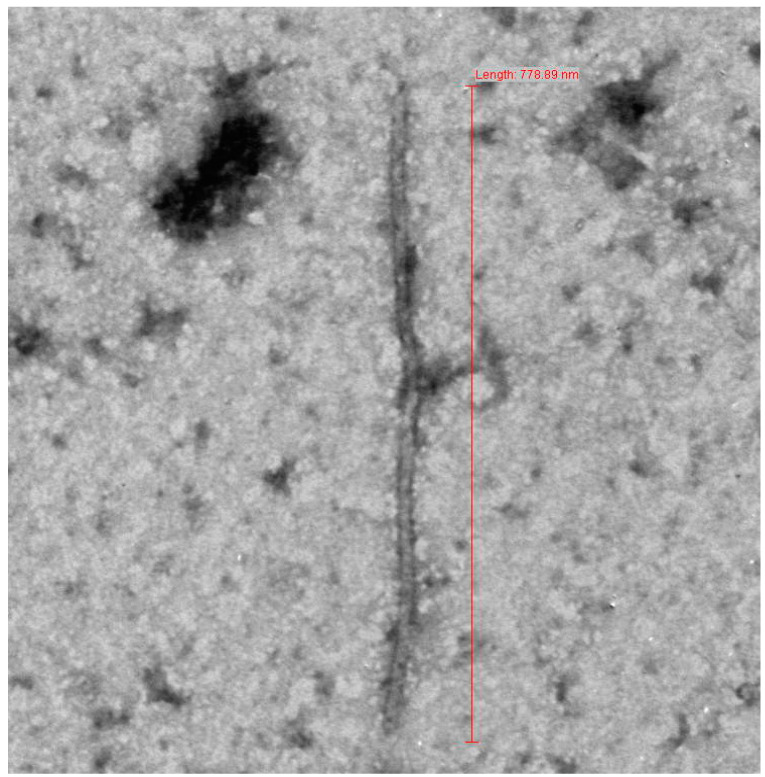
Transmission electron micrograph of symptomatic leaves tuberose.

Tuberose is an economically important, vegetatively propagated flower crop grown worldwide [13]. In 2021 and 2022, symptoms like chlorotic stripes in leaves that initiated from the base and yellowing of the whole leaf, characteristic of potyvirus infection were observed in tuberose plants grown at the Horticultural Research Centre, Sardar Vallabhbhai Patel University of Agriculture and Technology, Meerut. The disease incidence of 79% was recorded. To detect the presence of viral particles, twenty-four symptomatic leaf samples were collected and samples were observed under TEM, which revealed the presence of flexuous particles of length 778.89 nm (Figure 1). Morphology and size of the virus particle was similar to those of potyviruses [14]. An increasing number of viral isolates have been unraveled utilizing HTS around the world, allowing for major advancements in diagnostic tools [6,7,8,9,10,11,12,15]. Furthermore, HTS technology may provide detailed insights into the etiological and epidemiological correlations of viruses associated with diseased plant samples [8,9,10,11,12,15,16]. Thus, HTS was employed to identify the viral agent causing the observed disease in the present study.

RNA of pooled symptomatic leaf samples was sequenced using the Illumina HiSeq 2000 platform to identify viruses that may be associated with the symptoms. In the two libraries, the Illumina sequencing data generated was approximately 90 million 75 bp paired-end reads and after trimming, 90,380,608 reads (average length 75 bp) were acquired (Table S1). A total of 108,668 contigs were generated from samples of tuberose. BLASTx search against the nr database revealed the complete-genome sequence of tuberose mild mosaic virus (TuMMV) and tuberose mild mottle virus (TuMMoV). Copy numbers of TuMMV and TuMMoV were 11,011 and 108,535, respectively. In public domain, only five partial sequences of TuMMV isolates deposited from Taiwan, India, and USA [17,18], and eight partial sequences of TuMMoV isolates deposited from China, India, Mexico, Netherlands, and Taiwan [19,20] are available up to date.

In present study, the obtained TuMMV and TuMMoV genomes contained 9485 and 9462 nt, respectively excluding the poly(A) tail. TuMMV and TuMMoV genomic RNA contained a large ORF, encoding for a polyprotein of 3070 aa and 3081 aa, which began with an ATG codon at 138 and 164 nt and ended with UAG and UAA termination codon at 9350 and 9490 nt, respectively. The characteristic domains, conserved cleavage sites, and motifs specific to the genus *Potyvirus* [21,22,23] were predicted in the polyprotein sequence encoded by the genomes.

Furthermore, a PIPO ORF resulting from viral RNA polymerase slippage in the TuMMV and TuMMoV P3 cistrons was predicted downstream of the G_1-2_A_6–7_ motif at positions 2604–2859 nts and 2625–2888 nts, respectively. This motif resembled the highly conserved G_1-2_A_6–7_ motif found in other members of the family *Potyviridae* [24]. Like other potyviruses [25], TuMMV polyprotein is proteolytically processed into 10 mature proteins: P1, HC-Pro, P3, 6K1, CI, 6K2, NIa-VPg, NIa-Pro, Nib, and CP, at aa positions 298, 756, 1099, 1151, 1787, 1841, 2033, 2276, and 2798, respectively (Table S2). Similarly, TuMMoV polyprotein is proteolytically cleaved to yield 10 mature proteins: P1, HC-Pro, P3, 6K1, CI, 6K2, NIa-VPg, NIa-Pro, Nib, and CP, at aa positions 299, 757, 1100, 1152, 1788, 1841, 2033, 2276, and 2797, respectively (Table S3).

Furthermore, potyvirus-specific conserved motifs were discovered in proteins encoded by the genomes of both the viruses. The predicted motifs in TuMMV cleavage products are ^256^H-(X)_8_-D-(X)_33-34_-S^298^ in P1; ^349^K-I-T-C^352^ (aphid transmission), ^494^C-D-N-Q-L-D^499^ (symptomatology), and ^607^P-T-K^609^ (aphid transmission) in HC-Pro; ^1455^V-A-T-N-I-I-E-N-G-V-T-L^1466^ (potential helicase activity) and ^1506^G-R-V-G-R^1510^ in CI; ^1237^G-(X)_2_-G-X-G-K-S^1244^, ^2466^F-T-A-A-P^2470^, ^2480^C-V-D-D^2483^, ^2584^G-N-N-S-G-Q-P-S-T-V-V-D-N-S-L-M-V^2600^ (RNA-dependent polymerase activity), ^26288^GDD^2630^ (RNA-dependent polymerase activity), and ^2670^W-F-M-S^2673^ in NIb; ^2817^DAG^2819^ (aphid transmission) and ^3023^QMKAAA^3028^ in CP. Similarly, in TuMMoV, the predicted motifs in mature proteins are ^206^H-(X)_8_-D-(X)_33-34_-S^250^ in P1; ^350^K-I-T-C^353^ (aphid transmission), ^495^C-D-N-Q-L-D^500^ (symptomatology) in HC-Pro; ^1456^V-A-T-N-I-I-E-N-G-V-T-L^1467^ (potential helicase activity) and ^1507^G-R-V-G-R^1511^ in CI; ^2466^F-T-A-A-P^2470^, ^2480^C-V-D-D^2483^, ^2584^G-N-N-S-G-Q-P-S-T-V-V-D-N-S-L-M-V^2600^ (RNA-dependent polymerase activity), ^2628^GDD^2630^ (RNA-dependent polymerase activity), and ^2670^W-F-M-S^2673^ in NIb; ^2817^DAG^2819^ (aphid transmission) and ^3033^QMKAAA^3038^ in CP [26,27]. Few of these motifs are suggested to play critical roles in the transmission of potyviruses by aphids [28]. Despite the presence of all of these conserved motifs, aphid transmission necessitates species-specific interactions [29,30]. Further researches are needed to determine the aphid species transmitting TuMMV and TuMMoV.

In Blast analysis, coding-complete genomic RNA sequence of TuMMV shared 72.40–76.80% sequence identities with complete genomes of other potyviruses like pokeweed mosaic virus (KU133475), plum pox virus (OK562680), turnip mosaic virus (AB093615), leek yellow stripe virus (AJ307057), Japanese yam mosaic virus (KJ789139), Moroccan watermelon mosaic virus (MN688647), sunflower chlorotic mottle virus (GU181200), and sweet potato feathery mottle virus (MH763680) while that of TuMMoV shared 67.95–77.00% sequence identities with other viruses like Pennisetum mosaic virus (JX070146), turnip mosaic virus (AB093615), leek yellow stripe virus (AJ307057), jasmine virus T (KX398054), and achyranthes bidentata mosaic virus (MT648692). Pairwise sequence identities (%) of the coding-complete genomic RNA sequence of TuMMV (ON116187) shared nucleotide (nt) identities 48.10–59.0% and amino acid (aa) identities 41.30–61.80%, and TuMMoV (and ON219793) shared nt identities 47.40–59.0% and aa identities 42.0–62.30% with some other closely related potyvirus isolates reported globally (Table 1). According to ICTV species demarcation, these results indicate that they belong to the same species [3,4]. Since 76% nt sequence identity in the complete genome has been defined as the species demarcation criteria for potyviruses [31], the Indian isolates of TuMMV and TuMMoV can be considered members of a distinct potyvirus species.

**Figure 2:**
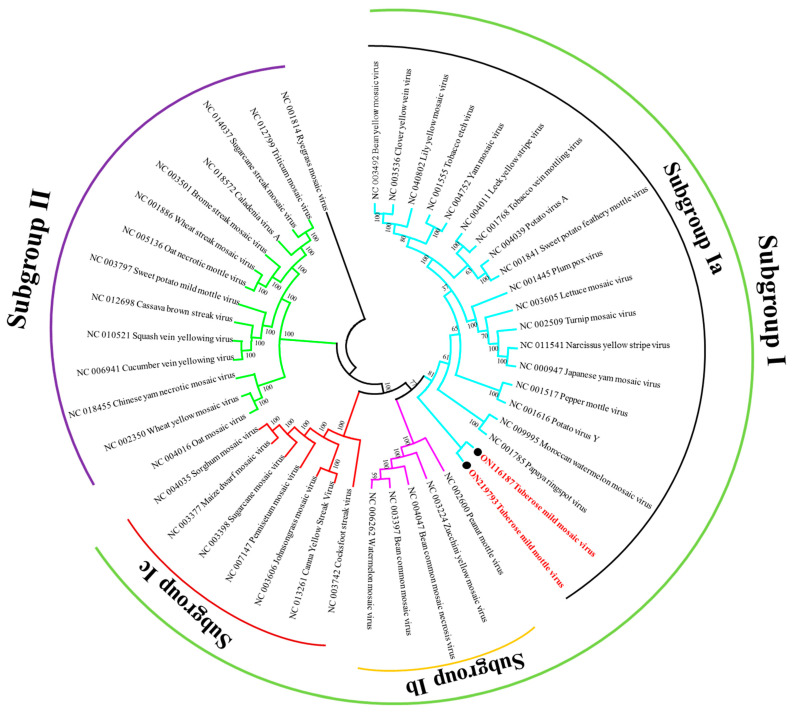
Complete genome-based phylogenetic tree using the neighbor joining algorithm with p-distance matrix and 1000 bootstrap replicates. The scale bar represents a genetic distance of 0.1. Viral genome sequences of the current study is highlighted in red. Ryegrass mosaic virus was used as an outgroup.

To analyze the evolutionary relationship between TuMMV, TuMMoV, and 44 other potyviruses, a phylogenetic tree based on their complete genomes was constructed. The results showed that these potyviruses can be divided into two distinct groups, subgroup I and subgroup II. Further, subgroup I divided into three distinct groups, subgroup Ia, subgroup Ib, and subgroup Ic. TuMMV and TuMMoV genomes that shared 65.20% nt identity clustered together in a single subgroup Ia, and both the viruses were showed genetic relatedness with papaya ring spot virus and Moroccan watermelon mosaic virus (Figure 2).

**Figure 3:**
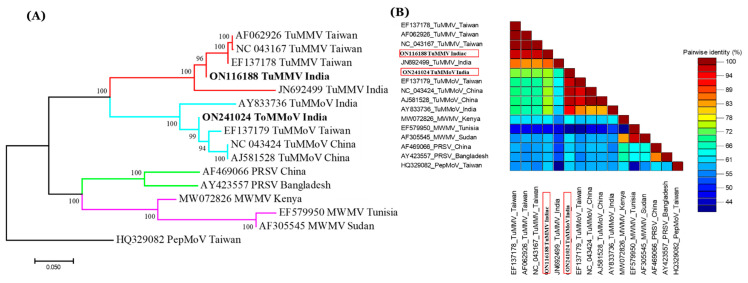
Phylogenetic analysis of TuMMV and TuMMoV based on partial coat protein sequences. (**A**) Phylogenetic tree of TuMMV and TuMMoV isolates and other viruses worldwide were constructed using partial CP sequences using neighbour-joining algorithm and p-distance matrix with 1000 bootstrap replicates. (**B**) Pairwise identity plots of partial CP sequences of TuMMV and TuMMoV aligned by Clustal W and visualized using Sequence Demarcation Tool software. Colors on the scale denote the pairwise identities on colored heat map. Partial CP sequences of the current study is highlighted in red.

RT-PCR assay using primers mentioned in Table 1 yielded an expected amplicon of ~700 bp (Figure S1) from 31 samples representing four cultivars, including samples used for HTS. The sequences obtained were deposited to NCBI GenBank with accession numbers ON116188-ON116195 and ON241024–ON241026. BLASTn analysis of the partial CP gene sequence of TuMMV showed 87.33–97.31% nt sequence identity with other TuMMV isolates available in GenBank while partial CP TuMMoV sequences of this study shared 87.33–97.31% nt identities with other TuMMoV isolates available in GenBank. Pairwise sequence identity comparison of the partial CP sequence of TuMMoV with similar sequences shared 95.80–98.40% nt and 96.0–99.56% aa identities with the global isolates (Figure 3B). The TuMMV isolate shared highest sequence identities (97.33% nt and 96.7% aa) with a Taiwanian (EF137178) isolate while TuMMoV shared highest sequence identities (98.40% nt and 99.56%) with a Chinese isolate (AJ581528) whereas the suggested threshold for species demarcation in the coat protein is 76% [3,4].

To better understand the genetic variability of the TuMMV isolates, a phylogenetic tree was constructed based on the partial CP coding sequences of TuMMV and TuMMoV from different geographical locations. In the phylogenetic tree, the TuMMV isolate of this study fell in sister clade to the TuMMV Taiwanian isolates while the TuMMoV isolate obtained in the current study formed a sister clade to the TuMMoV isolates of China (Figure 3A).

Phylogenetic analysis based on the genomic RNA sequence (Figure 2) and partial coat protein region (Figure 3) showed consistent clustering of isolates suggesting a close relationship between the Indian isolate and other TuMMV and TuMMoV isolates, supported by high posterior probability values.

## 3. Materials and Methods

### 3.1. Sample Collection and Electron Microscopy

Symptoms of viral disease were observed in the experimental field of tuberose cv. Mexican-single, Hyderabad-single, Rajni-phule, and Sikkim-selection during January 2021 at the Horticulture Research Center, Sardar Vallabhbhai Patel University of Agriculture and Technology, Meerut, India. Leaf samples exhibiting typical symptoms of viral diseases such as mosaic, puckering, and stunting were collected and stored at −80 °C till further analysis along with asymptomatic samples. Sap obtained from symptomatic leaf samples were examined under transmission electron microscope (TEM) after staining with 2% aqueous uranyl acetate (UA) by following the leaf dip method [32].

### 3.2. RNA Extraction, Library Preparation and HTS

Leaf samples of Mexican-single and Hyderabad-single cultivar were subjected to total RNA extraction using GeneJET RNA purification kit (Thermo Scientific, MA, USA) in accordance with the user instructions manual. The quality and quantity of total RNA were assessed using Qubit™ fluorometer (Thermo Fisher Scientific, MA, USA) and TapeStation (Agilent, CA, USA). From the isolated pool of total RNA, ribosomal RNA was depleted using the Ribo-Zero rRNA Removal Kit (Illumina, San Diego, CA, USA). Sequencing libraries were prepared from the ribodepleted RNA pool using truseq-stranded-total RNA library preparation kit (Illumina, CA, USA) by following the manufacturer’s instructions. Bioanalyzer (Agilent, CA, USA) and TapeStation were used to verify the quality of the prepared libraries and the libraries were sequenced using Illumina HiSeq 2000 platform at NxGenBio Life Sciences (New Delhi, India) to produce 2 × 75 bp paired-end reads.

### 3.3. Data Processing and Virus Analysis

Raw reads obtained were imported into CLC Genomic workbench (20.0.4) and quality trimmed using the trimming tool to remove ambiguous and adaptor sequences. Trimmed reads were assembled using the de novo assembly tool in CLC Genomic workbench (20.0.4) to obtain longer contigs. Obtained contigs were subjected to BlastX analysis against non-redundant (NR) database in the workbench. The alignment against reference viral genomes in the database were made using OmicsBox 2.1 (https://www.biobam.com/omicsbox, accessed on 23 February 2022). The outcomes showed in a report have a number of assembled reads and total used reads. Open reading frames (ORFs) encoded by the putative viral genomes were determined by NCBI ORF Finder (https://www.ncbi.nlm.nih.gov/orffinder/, accessed on 2 March 2022). Conserved domains in the virus proteins were predicted using NCBI Conserved Domain-Search tool (https://www.ncbi.nlm.nih.gov/Structure/cdd/wrpsb.cgi, accessed on 2 March 2022), and the genome organization of the identified viruses was constructed using the Bioedit 7.2 program [33].

### 3.4. Sequence Similarity and Phylogenetic Analysis

Genome sequences of identified viruses along with the available genome sequences of known potyviruses were retrieved and subjected to multiple sequence alignment using the CLUSTALW tool in MEGA-X [34]. Phylogenetic trees were constructed using neighbour-joining method and 1000 bootstrap replicates in MEGA-X. The nucleotide and amino acid sequence compositions and percent sequence identities were calculated using Bioedit 7.2 program [33] and visualized by the Sequence Demarcation Tool version 1.2 [35].

### 3.5. RT-PCR and Sanger Sequencing

Total RNA isolation was performed from four tuberose cvs. Mexican-single, Hyderabad-single, Rajni-phule, and Sikkim-selection as described in Section 3.2. cDNAs were synthesized from isolated RNA using random hexamers (1 µL) and RevertAid Reverse Transcriptase kit (Thermo Scientific, Waltham, MA, USA) according to the manufacturer’s protocol. PCR amplification of identified viruses was performed using virus-specific primers designed from the coat protein (CP) region of the obtained viral genomes (Table S4). The final 25 µL reaction volume contained 1x reaction buffer, 10 µM of each forward and reverse primers and 12.5 µL of DreamTaq PCR MasterMix (Thermo Scientific, Waltham, MA, USA). The reaction conditions were as follows: initial denaturation at 94 °C for 10 min, 35 cycles of 94 °C for 30 s, 52/55 °C for 45 s, 72 °C for 1 min followed by final extension at 72 °C for 10 min. PCR amplicons were purified and subjected to bidirectional Sanger sequencing at Centyle Biotech Pvt., Ltd., New Delhi, India.

## 4. Conclusions

Virus diseases limit tuberose production worldwide. With increased global trade, the possibility of introduction of tuberose viruses into newer areas grows by the day. As a result, rapid and reliable tuberose virus detection techniques are required to halt this inadvertent introduction and ensure virus-free tuberose production. We used HTS in this study and determined the first coding-complete genome sequences of TuMMV and TuMMoV. The genome sequences would be useful in providing a clearer picture of the virus evolution and in inferring biological properties of the viruses, which ultimately would help in devising management strategies. However, biological studies are required to understand the mode of transmission and effects of single/mixed viral infection on tuberose plants.

## Figures and Tables

**Table 1:** Comparisons of nucleotide sequence (nts) and amino acid (aa) identity of pairwise combinations of complete genomic RNA sequences of TuMMV and TuMMoV with other potyviruses.

Sequence Identity nts/aa	ON116187 Tuberose mild mosaic	ON219793 Tuberose mild mottle virus	NC001785 Papaya ringspot virus	NC009995 Moroccan watermelon mosaic virus	NC001616 Potato virus Y	NC001517 Pepper mottle viru	NC000947 Japanese yam mosaic virus	NC011541 Narcissus yellow stripe virus	NC002509 Turnip mosaic virus	NC003605 Lettuce mosaic virus	NC001445 Plum pox virus	NC001841 Sweet potato feathery mottle virus	NC004039 Potato virus A	NC001768 Tobacco vein mottling virus	NC004011 Leek yellow stripe virus	NC004752 Yam mosaic virus	NC001555 Tobacco etch virus	NC040802 Lily yellow mosaic virus	NC003536 Clover yellow vein virus	NC003492 Bean yellow mosaic virus
ON116187 Tuberose mild mosaic virus	ID	71.0	61.8	46.0	43.0	43.3	45.4	45.1	45.4	41.7	45.7	42.8	43.6	43.7	41.3	44.5	43.4	42.6	42.0	43.2
ON219793 Tuberose mild mottle virus	65.2	ID	62.3	46.2	43.1	43.3	44.4	44.9	45.0	42.0	45.1	42.3	43.7	43.9	41.7	43.6	43.3	43.0	43.0	43.5
NC001785 Papaya ringspot virus	58.1	57.9	ID	57.9	41.0	41.0	42.8	42.3	42.5	43.0	44.0	42.3	41.9	41.3	44.0	42.2	49.0	42.3	47.0	41.3
NC009995 Moroccan watermelon mosaic virus	49.5	49.0	61.0	ID	43.8	43.7	45.0	45.3	45.8	43.8	47.6	44.3	43.8	44.1	42.9	45.8	44.3	44.1	43.9	44.5
NC001616 Potato virus Y	54.0	55.0	48.1	49.1	ID	61.3	44.8	45.3	45.9	45.6	48.0	43.5	44.7	45.2	42.0	47.2	45.7	43.2	42.8	43.8
NC001517 Pepper mottle virus	59.0	59.0	47.8	48.5	61.7	ID	45.2	45.7	46.3	45.0	47.6	43.6	44.6	44.3	42.1	46.3	45.8	43.3	43.4	43.7
NC000947 Japanese yam mosaic virus	50.0	48.8	49.7	49.9	49.5	49.9	ID	58.8	58.6	46.9	50.1	46.9	47.0	46.8	43.4	48.1	46.3	45.2	45.2	46.1
NC011541 Narcissus yellow stripe virus	49.2	48.3	48.7	49.3	49.8	49.2	61.9	ID	60.8	46.3	50.8	47.6	47.5	46.8	43.7	47.3	46.4	45.6	45.9	45.9
NC002509 Turnip mosaic virus	49.2	48.6	49.0	51.0	49.9	49.9	62.0	63.0	ID	47.4	50.9	49.3	48.5	46.8	44.1	47.8	46.8	45.3	45.2	45.1
NC003605 Lettuce mosaic virus	47.9	47.4	49.0	49.2	49.3	48.4	54.1	53.5	54.1	ID	47.7	45.3	44.1	43.9	42.4	46.1	43.7	42.7	43.0	42.9
NC001445 Plum pox virus	59.0	50.0	51.0	51.2	51.1	59.0	52.9	51.7	52.5	51.4	ID	50.3	49.1	47.0	46.2	50.3	47.3	47.8	46.4	47.4
NC001841 Sweet potato feathery mottle virus	48.9	48.9	54.0	56.0	49.1	49.7	51.4	51.4	51.1	54.0	54.2	ID	45.6	43.6	42.4	46.0	44.2	43.6	42.9	44.0
NC004039 Potato virus A	48.4	49.3	48.5	49.4	49.1	48.5	58.0	51.3	51.1	49.6	52.2	55.8	ID	54.6	43.5	46.9	48.5	45.8	45.5	46.4
NC001768 Tobacco vein mottling virus	49.6	49.4	47.8	48.3	53.0	49.2	49.1	49.1	48.7	48.9	49.8	49.6	52.1	ID	43.9	46.7	49.3	45.4	44.8	44.9
NC004011 Leek yellow stripe virus	49.6	49.2	48.7	48.5	49.9	49.7	49.4	48.6	49.3	48.5	51.1	49.6	49.3	54.6	ID	44.0	43.2	47.6	45.0	45.7
NC004752 Yam mosaic virus	48.8	47.9	49.0	49.5	49.1	49.0	57.0	49.4	54.0	49.3	52.0	51.5	51.3	48.9	48.5	ID	45.4	44.9	45.7	45.1
NC001555 Tobacco etch virus	48.4	47.7	47.6	48.6	49.3	48.7	49.8	49.3	49.7	48.6	51.2	58.0	51.6	49.8	48.9	54.8	ID	44.8	45.2	45.6
NC040802 Lily yellow mosaic virus	48.7	48.1	49.0	49.5	48.8	49.3	52.0	49.4	49.9	49.3	52.6	51.0	58.0	48.3	53.0	52.0	51.2	ID	49.3	49.4
NC003536 Clover yellow vein virus	48.8	48.2	48.7	49.4	48.6	49.2	50.0	49.5	56.0	48.6	51.4	53.0	58.0	48.5	49.7	51.9	52.2	55.8	ID	68.9
NC003492 Bean yellow mosaic virus	48.3	48.1	49.0	49.1	49.3	49.4	49.8	49.6	49.6	48.7	51.4	53.0	59.0	48.6	53.0	52.1	51.7	54.9	65.9	ID

## Data Availability

The HTS data used in this study have been deposited under the NCBI SRA under Bioproject: PRJNA810951 (SRA accessions: SRR18162840 and SRR18162840). The complete genomic RNA sequence of TuMMV and TuMMoV have been deposited in NCBI under accession number ON116187 and ON219793, respectively. Partial CP gene sequences have been deposited under accession numbers ON116188-ON116195 and ON241024-ON241026.

## Supplementary Materials

The following supporting information can be downloaded at: https://www.mdpi.com/article/10.3390/pathogens11080861/s1. Table S1: HTS statistics for each sample before and after quality control showing the number of reads and the range of read lengths in base pairs (bp), Table S2: Putative genes along with nine protease cleavage sites in polyprotein of TuMMV, Table S3: Putative genes along with nine protease cleavage sites in polyprotein of TuMMoV, Table S4: List of virus-specific primers designed from the coat protein (CP) region of the obtained viral genomes, Figure S1: PCR detection of TuMMV and TuMMoV. Expected amplicon size was ~700 bp. Lane M, 1 kb DNA ladder (Cat. No. SM0311; Thermo Scientific); Lanes 2, 3, 4, 5, 6, 7, 8, 9, 10 and 11, PCR amplified products of TuMMV; Lane 12, 13, 14, 15, 16, 17, 18, 19, 20, 21 and 22, PCR amplified products of TuMMoV.
