# Supplementary material for: Complete Genomic RNA Sequence of Tuberose Mild Mosaic Virus and Tuberose Mild Mottle Virus Acquired by High-Throughput Sequencing
Source: Pathogens. 2022 Jul 30;11(8):861. doi: 10.3390/pathogens11080861 (PMC9412269; doi:10.3390/pathogens11080861)
Supplement: Supplementary file 1 [file pathogens-11-00861-s001.zip › pathogens-1824987-supplementary.pdf]

Table S1. Summary of raw reads obtained through High-throughput Sequencing of diseased tuberose samples and trimming statistics

Sample	Input Reads	Average length	No. of read after Trimming	Percentage trimmed (%)	Average length after trim
**Mexican single**	53,374,692	75.48	53,370,490	99.99	75.47
**Hyderabad single**	37,005,916	75.45	37,000,244	99.98	75.44

Table S2. Putative genes along with nine protease cleavage sites in polyprotein of TuMMV.

Region	Start-End site	Size in nt/aa	Cleavage site (C-Terminus)
**5'UTR**	1-138	138/-	
**P1**	139-1032	894/298	Y/S
**HC-Pro**	1033-2406	1374/458	G/G
**P3**	2407-3435	1029/343	Q/I
**6K1**	3436-3591	156/52	Q/S
**CI**	3592-5499	1908/636	Q/A
**6K2**	5500- 5661	162/54	Q/G
**VPg**	5662- 6237	576/192	E/S
**NIa-Pro**	6238-6966	729/243	Q/G
**NIb**	6967-8532	1566/522	Q/S
**CP**	8533-9351	819/273	-
**3'-UTR**	9352-9485	134/-	-

Table S3. Putative genes along with nine protease cleavage sites in polyprotein of TuMMoV.

Region	Start-End site	Size in nt/aa	Cleavage site (C-Terminus)
**5’UTR**	1-164	164/-	
**P1**	165-1061	897/299	Y/S
**HC-Pro**	1062-2435	1374/458	G/G
**P3**	2436-3464	1029/343	Q/A
**6K1**	3465-3620	156/52	Q/S
**CI**	3621-5528	1908/636	Q/S
**6K2**	5528-5687	159/53	Q/G
**VPg**	5688-6263	576/192	E/S
**NIa-Pro**	6264-6992	729/243	Q/S
**NIb**	6993-8555	1563/521	Q/S
**CP**	8556-9409	855/285	-
**3’-UTR**	9410-9462	53/-	-

Table S4: List of virus-specific primers designed from the coat protein (CP) region of the obtained viral genomes.

	Primer Sequence	Product Length
TuMMV	**F:** 5’-AAGATGATGTCAACAATGCA-3’	~700 bp
	**R:** 5’-TTACGCACTCCCAACAAAG-3’	
TuMMoV	**F:** 5’-TCCGTAGGTGAACCTGCGG-3’	~700 bp
	**R:** 5’-TCCTCCGCTTATTGATATGC-3’	

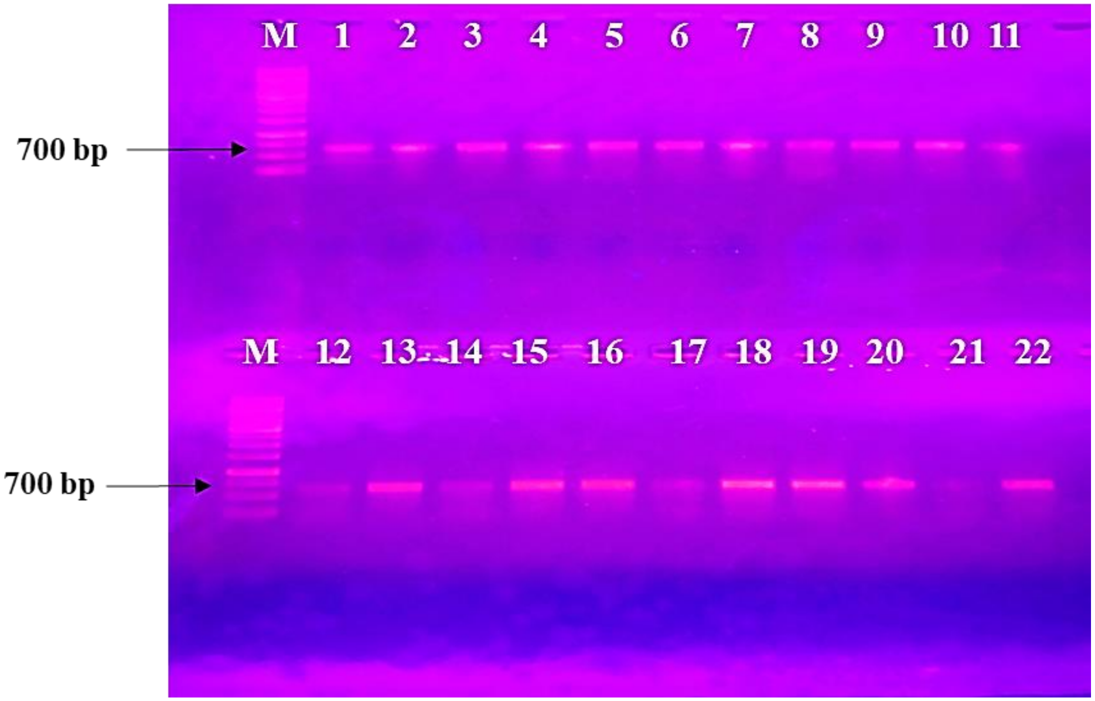

Figure S1. PCR detection of TuMMV and TuMMoV. Expected amplicon size was ~700 bp. Lane M, 1 kb DNA ladder (Cat. No. SM0311; Thermo Scientific); Lanes 2, 3, 4, 5, 6, 7, 8, 9, 10 and 11, PCR amplified products of TuMMV; Lane 12, 13, 14, 15, 16, 17, 18, 19, 20, 21 and 22, PCR amplified products of TuMMoV.
